# Integrating Remote Sensing Information Into A Distributed Hydrological Model for Improving Water Budget Predictions in Large-scale Basins through Data Assimilation

**DOI:** 10.3390/s8074441

**Published:** 2008-07-29

**Authors:** Changbo Qin, Yangwen Jia, Z.(Bob) Su, Zuhao Zhou, Yaqin Qiu, Shen Suhui

**Affiliations:** 1 Department of Water Resources, Institute of Water Resources and Hydropower Research (IWHR), Beijing, 100038, China; 2 International Institute for Geo-Information Science and Earth Observation (ITC), 7500AA Enschede, The Netherlands; 3 The Center for Clean Technology and Environmental Policy (CSTM), University of Twente, 7500AE Enschede, The Netherlands

**Keywords:** Evapotranspiration, Distributed hydrological model, Data assimilation, WEP, SEBS, Extended Kalman Filter

## Abstract

This paper investigates whether remote sensing evapotranspiration estimates can be integrated by means of data assimilation into a distributed hydrological model for improving the predictions of spatial water distribution over a large river basin with an area of 317,800 km^2^. A series of available MODIS satellite images over the Haihe River basin in China are used for the year 2005. Evapotranspiration is retrieved from these 1×1 km resolution images using the SEBS (Surface Energy Balance System) algorithm. The physically-based distributed model WEP-L (Water and Energy transfer Process in Large river basins) is used to compute the water balance of the Haihe River basin in the same year. Comparison between model-derived and remote sensing retrieval basin-averaged evapotranspiration estimates shows a good piecewise linear relationship, but their spatial distribution within the Haihe basin is different. The remote sensing derived evapotranspiration shows variability at finer scales. An extended Kalman filter (EKF) data assimilation algorithm, suitable for non-linear problems, is used. Assimilation results indicate that remote sensing observations have a potentially important role in providing spatial information to the assimilation system for the spatially optical hydrological parameterization of the model. This is especially important for large basins, such as the Haihe River basin in this study. Combining and integrating the capabilities of and information from model simulation and remote sensing techniques may provide the best spatial and temporal characteristics for hydrological states/fluxes, and would be both appealing and necessary for improving our knowledge of fundamental hydrological processes and for addressing important water resource management problems.

## 1. Introduction

Because water is becoming the limiting factor for development in many parts of the world, more systematic approaches are needed to analyze the uses, depletion, and productivity of water. An improved knowledge of the land surface hydrologic states and fluxes, and of their spatial and temporal variability across different scales, is urgently needed in many hydrologic studies and water resources management [1-2]. At present many tools can help for water budget analysis, including distributed models, geographic information systems (GIS) and remote sensing techniques.

A great variety of distributed hydrological models have been developed, ranging from simple empirical equations, to complex systems of partial differential equations, which can incorporate the spatial distribution of various inputs and boundary conditions, such as topography, vegetation, land use, soil characteristics, rainfall, and evaporation, and produce spatially detailed outputs such as soil moisture, water table, groundwater fluxes, and surface saturation patterns. However, distributed modeling of hydrological processes has its limitations. The major problems are over-parameterization and uncertainty, in the sense that most models have not been validated in all their details. New data sources for observation of hydrological processes can alleviate some of the problems facing the validation and operational use of hydrological models. Satellite, airborne and ground-based remote sensing has begun to fulfill some of its potential for hydrological applications, allowing monitoring and measurement of rainfall, snow, soil moisture, vegetation, surface temperature, energy fluxes, and land cover over large areas. The main reason is that remote sensing data can provide large-scale, systematic land surface observations consistently over the large scale [3].

The integration of data and models is referred to as data assimilations (DA) which provides a means of integrating data in a consistent manner with model predictions and merge measurements of different types, accuracies, and resolutions into spatially distributed models [4-5]. For example, remotely sensed observations and land surface modeling have been integrated in both NASA's Global Water and Energy Cycle (GWEC) program, and the World Climate Research Cycle (GWEC) program, and the World Climate Research Programme's (WCRP) Global Energy and Water Experiment (GEWEX).

The fundamental operative unit for water resources management is the catchment or river basin. Combining and integrating the capabilities and information within an integrated framework from simulation and remote sensing techniques is thus both appealing and necessary for improving our knowledge of fundamental hydrological processes and for supporting water resources management in the catchment or basin scale. Therefore, it's urgent to investigate appropriate DA and modeling approaches at the catchment/river basin scale, and more case studies should be conducted to realize the operational potential of DA for a broad range of water resources management problems. Therefore, the work presented in this paper is motivated by the need to develop an improved data assimilation system that use remote sensed ET to improve the predictive performance of a distributed hydrological model in a large river basin.

Many studies in hydrological modeling of DA have begun to appear in recent years [e.g. 6-22], spurred by the success of DA in other fields. Most of these studies focus on the assimilation of soil moisture data in land surface models, and rely on synthetic datasets to assess the performance of the DA algorithms. Much less work has been published on assimilating remote sensed evapotranspiration (ET)/latent flux (LE) into hydrological models at the regional/basin scale. Schuurmans et al. [23] address the question of whether remotely sensed latent heat flux estimates from Surface Energy Balance Algorithm for Land (SEBAL) over a catchment can be used to improve distributed hydrological model computations using a constant gain Kalman filter data assimilation algorithm in the Drentse Aa catchment with an drainage area of 300 km^2^. Pan et al. [3] proposed and tested a data assimilation system that consisted of a combination of two filters - a particle filter (PF) [24-25] and an ensemble Kalman filter (EnKF) [26-27] to estimate the water budget using a MODIS based estimate of surface evapotranspiration (ET) over the spatial domain of Red-Arkansas river basin.

## 2. A data assimilation system for water budget predictions

### 2.1 Overview

**Figure1:**
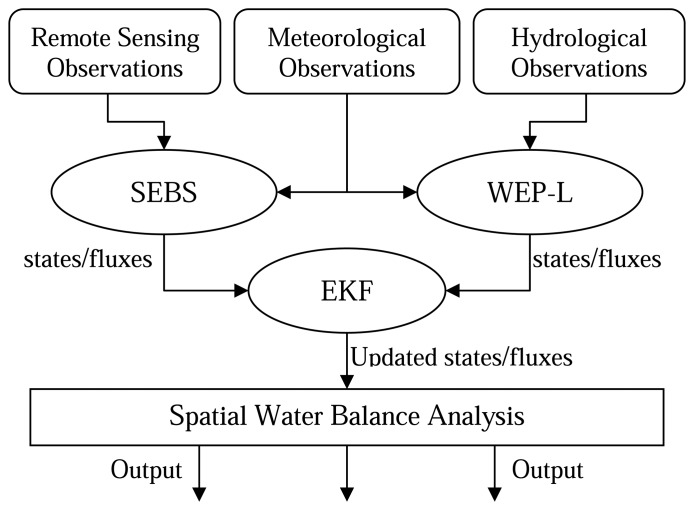
Data assimilation system scheme

The scheme of the data assimilation system is sketched in Figure 1. In this paper a series of MODIS satellite images available for the Haihe basin for the year 2005 are used. Evapotraspiration is retrieved from these 1×1 km resolution images using the SEBS (Surface Energy Balance System) algorithm [28]. The physically-based distributed model WEP-L (Water and Energy transfer Process in Large river basins) [29] is used to compute the water balance of the Haihe River basin in the same year. An extended Kalman filter (EKF) data assimilation algorithm, suitable for non-linear problems, is used.

In order to attain the water budgets for water resources assessment and planning, the specific setup of the WEP-L model is made to provide an accurate estimate of the magnitude of the different components of the hydrologic cycle in large basins like Haihe River basin. ET is an important component for water budget analysis, because ET can be regard as the net consumption of water. Remote sensing algorithm (e.g. SEBS) can provide spatially distributed estimates of evapotranspiration, although continuous time series with time steps are difficult. It is expected that the WEP-L model can benefit by assimilating the spatial distributed ET estimates provided by the SEBS, and give a better understanding about how the availability of actual evapotranspiration varies both spatially and temporally. The physical models, remote sensing retrieval tool, data assimilation techniques and data sources are further discussed below.

### 2.2 Description of the WEP-L model

With the computational resources available today to most modelers, it has become feasible to build and apply highly complex distributed hydrological models that represent many different processes and consist of many model elements. The distributed hydrological model WEP-L was developed in a national key basic research project of China [29-31]. The WEP-L model is based on the WEP model [32-34] which has been successfully applied in several watersheds in Japan, Korean and China with different climate and geographic conditions [32, 34-39]. The WEP-L model adopts the contour bands as the calculation units to fit for large river basins and has been applied in the Yellow river basin in China. For details one is referred to Jia et al. [29-31].

**Figure 2.**
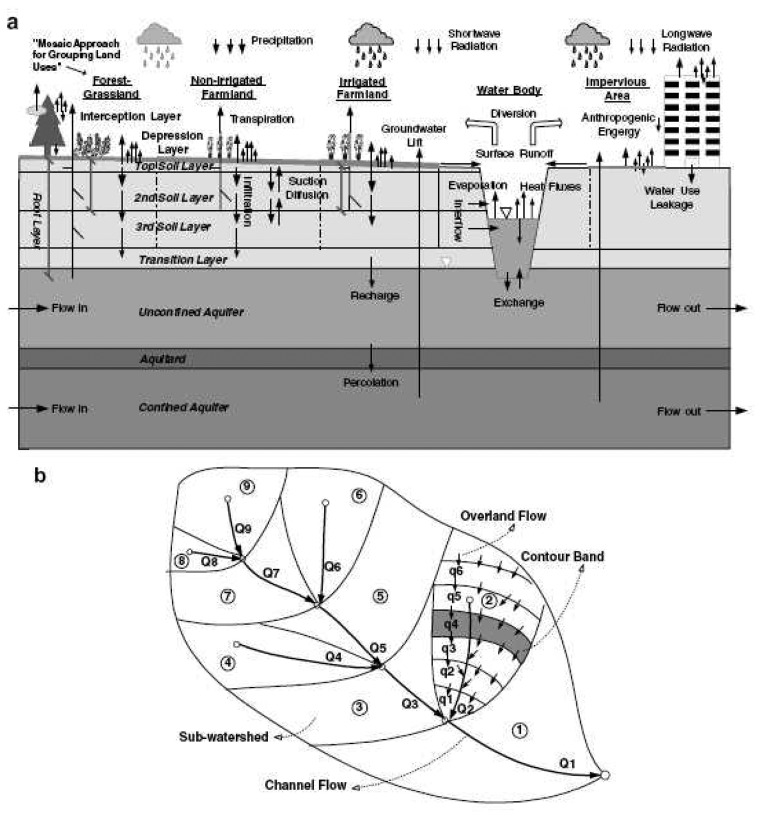
Schematic illustration of WEP-L model structure (Jia et al., 2006): **(a)** vertical structure within a contour band, and **(b)** horizontal structure within a sub-watershed.

The vertical structure of WEP-L within a contour band is shown in Figure 2(a), and the horizontal structure of WEP-L within a sub-watershed is shown in Figure 2(b). Land use is divided into five groups within a contour band, namely Soil Vegetation (SV) group, Non-irrigated farmland (NF) group, Irrigated Farmland (IF) group, Water Body (WB) group and Impervious Area (IA) group. The SV group is further classified into bare soil land, tall vegetation (forest or urban trees) and short vegetation (grassland). The IA group consists of impervious urban cover, urban canopy and rocky mountain. The areal average of water and heat fluxes from all land uses in a contour band produces the averaged fluxes in the contour band. For pervious groups of SV, NF and IF, nine vertical layers, namely an interception layer, a depression layer, three upper soil layers, a transition layer, an unconfined aquifer, an aquitard and a confined aquifer, are included in the model structure.

The simulated hydrological processes include snow melting, evapotranspiration, infiltration, surface runoff, subsurface runoff, groundwater flow, overland flow, river flow, and water use. The simulated energy transfer processes include short-wave radiation, long-wave radiation, latent heat flux, sensible heat flux, and soil heat flux. Adopted modeling approaches for hydrological and energy processes are referred to in Jia et al. [34](2001b) except snow melting and water use processes.

WEP-L integrates the merits of distributed hydrological models with those of SVATS (Soil-Vegetation-Atmosphere Transfer Schemes) model, couples the simulation of water cycle and energy processes, and calculates evapotranspiration from each land use separately. Evapotranspiration consists of interception of vegetation canopies (evaporation from the wet part of leaves), evaporation from water bodies, soil, urban cover and urban canopy and transpiration from the dry fraction of leaves with the source from the three upper soil layers. The evaporation from water bodies or ponded water in the depression storage is calculated with the Penman equation. The evaporation from the impervious area is taken as the smaller one of current depression storage and the potential evaporation. The computation of interception is referred to the Noilhan and Planton [40] model that is an interception reservoir method. The evaporation from soil is assumed to come only from the topsoil layer. The Penman equation is adopted to compute potential evaporation from which actual evaporation of soil is computed using a wetness function suggested by Lee and Pielke [41]. The actual transpiration is calculated using the Penman-Monteith equation [42] and the canopy resistance [40] which is related to the soil moisture condition. The average evapotranspiration in a contour band is obtained by areally averaging those from each land use.

### 2.3 Description of the SEBS algorithm

With the rapid development and increased application of remote sensing technology, evapotranspiration calculation methods using remote sensing techniques have become a major trend for hydrological research in recent years. The data obtained from visible, near-infrared and thermal band can reflect the spatial and temporal distribution of surface features, which have a great importance in simulating the energy balance. The SEBAL (Surface Energy Balance Algorithm for Land) proposed by Bastiaanssen et al. [43], which is based on land surface parameters acquired with remote sensing techniques. It is a semi-empirical model applied in calculating the evapotranspiration and the main difficulty is to determine the “hot” pixel and the “cold” pixel which has a great effect on the final results. Norman and Kustas [44] proposed the TSEB (Two-Source Energy Balance) algorithm to calculate the evaporation from bare soil and transpiration from vegetation separately based on remote sensing data.

The Surface Energy Balance System (SEBS) was developed by Su [28] to estimate the atmospheric turbulent fluxes and evaporative fraction using satellite earth observation data, in combination with meteorological information at proper scales. The system retrieves evapotranspiration (ET) using measurements of incoming surface radiation, surface skin temperature, surface meteorology, and surface and vegetation properties [45]. One of the advantages in this algorithm is applying both Bulk Atmospheric Similarity (BAS) and the Monin-Obukov atmospheric surface layer (ASL) similarity in the model, which can be used for regional and local scales respectively to determine the turbulent fluxes. Another important merit of SEBS is the inclusion of a physical model which takes surface heterogeneity into account in the estimation of the roughness height for heat transfer. The SEBS algorithm has been successfully applied for many applications of evaporation estimations in many different places with different scale [46-51].

For the purpose of this study, only basic equations for ET retrieval will be briefly described, full details are given by Su [28]. The surface energy balance is commonly written as

(1) $$R_{n}=G_{0}+H+\lambda E$$

Where *R_n_* is the net radiation, *G*_0_ is the soil heat flux, *H* is the turbulent sensible heat flux, and *λE* is the turbulent latent heat flux (*λ* is the latent heat of vaporization and *E* is the actual evapotranspiration).

To determine the evaporative fraction (to be defined below), use is made of energy balance considerations at limiting cases. Under the dry-limit, the latent heat (or the evaporation) becomes zero due to the limitation of soil moisture and the sensible heat flux is at its maximum value.

From Eqn. (1), it follows,

(2) $$\begin{array}{c} \lambda E_{\text{dry}}=R_{n}-G_{0}-H_{\text{dry}}\equiv 0,\text{or}\\ H_{\text{dry}}=R_{n}-G_{0} \end{array}$$

Under the wet-limit, where the evaporation takes place at potential rate, *λE_wet_*, (i.e. the evaporation is limited only by the energy available under the given surface and atmospheric conditions), the sensible heat flux takes its minimum value, *H_wet_*, i.e.

(3) $$\begin{array}{c} \lambda E_{\text{wet}}=R_{n}-G_{0}-H_{\text{wet}},\text{or}\\ H_{\text{wet}}=R_{n}-G_{0}-\lambda E_{\text{wet}} \end{array}$$

The relative evaporation then can be evaluated as

(4) $$\Lambda_{r}=1-\frac{H-H_{\text{wet}}}{H_{\text{dry}}-H_{\text{wet}}}$$

The evaporative fraction is finally given by:

(5) $$\Lambda =\frac{\lambda E}{R_{n}-G}=\frac{\Lambda_{r}\cdot \lambda E_{\text{wet}}}{R_{n}-G}$$

By inverting Eqn. (5), the actual latent heat flux *λE* can be obtained.

The actual sensible heat flux *H* is determined with the bulk atmospheric similarity approach and is constrained in the range set by the sensible heat flux at the wet limit *H_wet_*, and that at the dry limit *H_dry_*. *H_dry_* is given by Eqn. (2) and *H_wet_* can be derived by a combination equation [52] similar to the Penman–Monteith combination equation with the assumption of a completely wet situation. Other details are given in Su [28].

### 2.4 The extended Kalman filter

Data assimilation techniques can be used to improve model performance either by optimizing model parameters or by correcting the state produced by the model, both through a variational or a sequential approach. For operational purposes the combination of parameters optimization and state estimation is most promising. Nevertheless, in this paper we restrain ourselves to state estimation via a sequential approach, which provides a general framework to explicitly incorporate input, model and remote sensing observation errors. Among sequential data assimilation techniques, the Kalman filter (KF) introduced by Kalman [53] and originally devised for linear models, is the most widely used method. In order to perform DA on non-linear models, several different implementations of the KF have been devised: the Extended version (EKF) proposed by Jaswinski [54], the Ensemble version (EnKF) proposed by Evensen [27] and the Singular Evolutive Extended version (SEEK) proposed by Pham et al. [55].

A significant benefit of the Kalman filter is that unmeasured process states may be estimated based on limited, noisy process measurements. In this paper, the EKF is used for nonlinear systems that are linearized around the current process state, which take into account the estimated errors on the model and on the observations to determine the correction to be applied to the states and calculates a new estimation of these errors every time step. Thus, the more accurate the observations the closer the a posteriori state of the model will be to them.

In order to use EKF to perform parameter estimation, parameters are treated as process variables with a rate of change=0. We can define the state vector $\left(\begin{array}{c} x\\ \theta \end{array}\right)$ and observation vector z:

(6) $$\left(\begin{array}{c} \dot{x}\\ \theta \end{array}\right)=\left(\begin{array}{c} f(x,\theta ,t)\\ 0 \end{array}\right)+w(t),$$

(7) z=h(x,t)+v(t)

The evolution of the process state, *ẋ*, is a function of the state, *x*, time, *t*, and system parameters, *θ*. *w* and *v* are subject to zero mean Gaussian white noise functions. Their error covariance matrices represent the errors: *Q_k_* for the uncertainties on the model and *R_k_* for the uncertainties on the observations. *Q_k_* represents the errors generated by the model during one time step, and does not account for the total uncertainties of the model, which also include the propagation of uncertainties from *k*-1 to time *k*. Therefore, a matrix *P_k_* is defined to include the propagation of the former errors.

The heart of the Kalman filter is the gain matrix, *K*, which is calculated in two phases: the adjustment phase and the propagation phase.

During the first phase, the Kalman gain matrix *K_k_* is derived by the following formula:

(8) $$K_{k}=P_{k}^{-}\;H_{k}^{T}({\hat{x}}_{k}^{-}){\left[H_{k}\;({\hat{x}}_{k}^{-})P_{k}^{-}\;H_{k}^{T}\;({\hat{x}}_{k}^{-})+R_{k}\right]}^{-1}$$

Here *H* is the Jacobian matrix of *h* with respect to the observation vector. Eqn. (8) indicates that, for each state, the correction factor will be applied due to each observation and its calculation takes into account the error matrices *R_k_* and *P_k_*.

Then the state vector is updated: the a posteriori state vector ${\overset{⌢}{x}}_{k}$ is equal to the a priori state vector ${\overset{⌢}{x}}_{k}^{-}$ plus the difference between the observation and state vectors multiplied by the Kalman gain:

(9) $${\hat{x}}_{k}={\hat{x}}_{k}^{-}+K_{k}[z_{k}-h_{k}({\hat{x}}_{k}^{-})]$$

The a posteriori error covariance matrix *P_k_* is also updated as follows:

(10) $$P_{k}=[I-K_{k}H_{k}({\hat{x}}_{k}^{-})]P_{k}^{-}$$

During the second phase, the new error covariance matrix *P_k_*_+1_ is calculated by adding the error at time k *P_k_* propagated via the tangent linear and the error *Q_k_* generated during this time step, where *F* is the Jacobian matrix of *f* with respect to the state vector.

(11) $$P_{k+1}=F({\overset{⌢}{x}}_{k}^{-})P_{k}F^{T}({\overset{⌢}{x}}_{k}^{-})+Q_{k}$$

Calculation of the correction factor (Kalman gain) is an important step of the method: if this factor is too small, the assimilation procedure will have no effect, if it is too large, the model would forget its original evolution. The magnitude of the correction is dominated by the ratio of the errors on the observations and the model. If the uncertainties on the observations are very small (i.e. |*R_k_* / *P_k_*| ≈ 0), the Kalman gain will be almost equal to 1 and the states will set to the observations. Alternatively, if the observations have a large uncertainty, the Kalman gain will be almost equal to 0 and the correction will be almost null. The above expressions show that, if *Q_k_* is assumed to be zero, the magnitude of the error covariance will decrease and close to a value of zero. This would be true, only if the model is a perfect representation of reality, and the observations are perfect. However, we know that these assumptions cannot be made. So a certain level of uncertainties must be retained in the data assimilation process. A methodology to determine the optimal value for *R_k_* and *Q_k_* should be the focus of a further study, but falls outside the scope of this paper.

## 3. Description of study area

**Figure 3.**
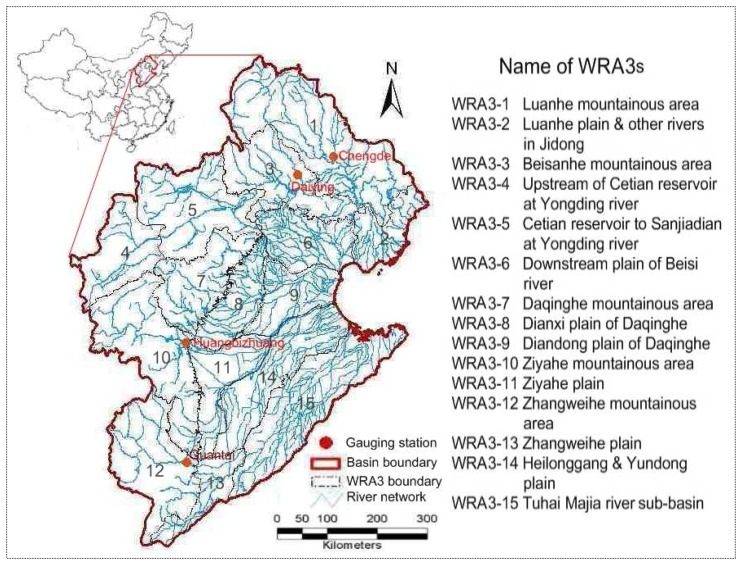
Map of Haihe River basin and name list of WRA3s.

The Haihe River basin is located between 35°∼43°N and 112°∼120E°. It neighbors the Inner Mongolian Plateau in the north, and the Yellow River is the borderline in the south. It faces the Bohai Sea to the east and borders Shanxi Plateau in the west. The Haihe basin belongs to the warm temperate zone with a semi-humid and semi-arid climate. The winters are dry and cold, with low rainfall in the spring and heavy rainfall in the summer. The average annual precipitation is 548 mm, about 80 percent of which falls during June to September. Its area is 317,800 square kilometers, of which 189,000 km^2^ is mountainous and the remainder is plain, and divided into 15 level-3 water resources areas (WRA3) (Figure 3). The Haihe River valley starts from the western Taihang Mountain and reaches the eastern Bohai Sea, running across Beijing City, Tianjin City, Hebei Province, Shanxi Province, Shandong Province, Henan Province, Liaoning Province and the Inner Mongolia Autonomous Region.

**Figure 4.**
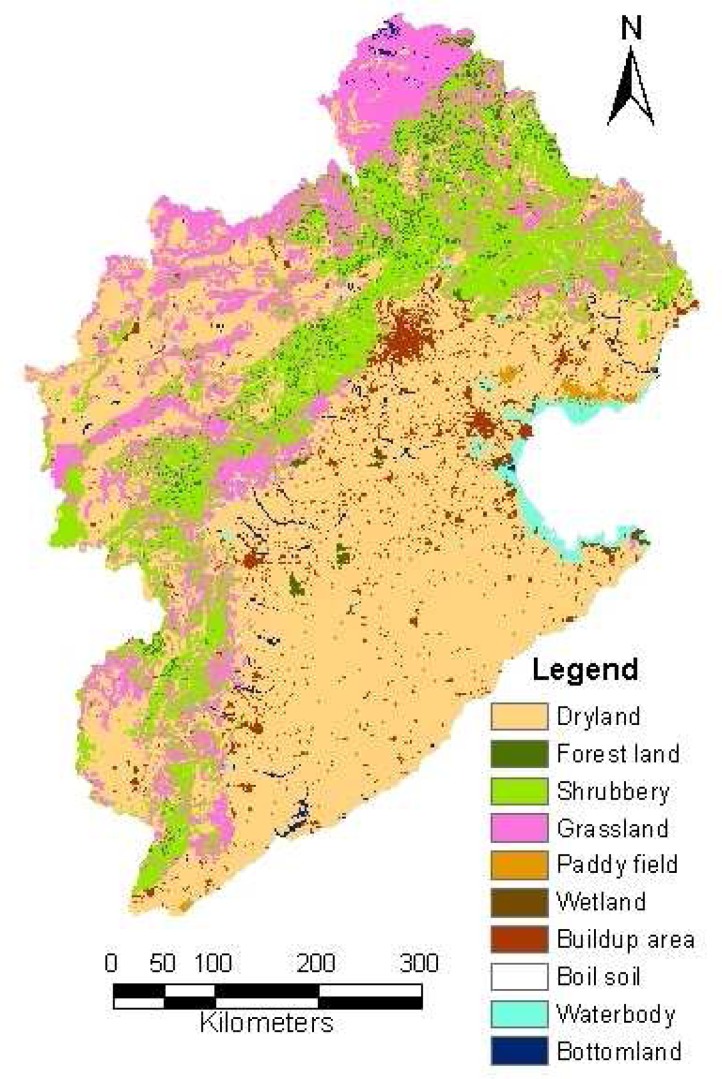
Land use patterns in the Haihe basin.

The main land use patterns in the Haihe River basin are dominated by dryland, forest land, shrubbery, grassland, paddy field, wetland, build-up area, bare soil, water body and bottomland (Figure 4).

## 4. Data preparation and assimilation results, discussion

### 4.1 WEP-L application to the Haihe basin

**Figure 5.**
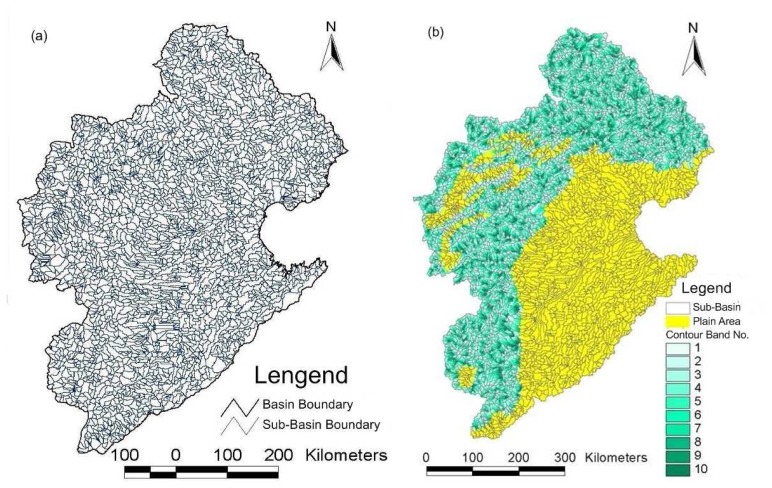
Subdivision of hydrological response units: **(a)** Subdivision of sub-basins; and **(b)** Subdivision of contour bands in mountainous area

The whole drainage basin of the Haihe basin in WEP-L model is divided into 3067 sub-watersheds (Figure 5(a)), each of which is assigned with a Pfafstetter code [56]. Each sub-watershed in hilly and tableland areas is further divided into 1-10 contour bands, but no further division is performed for sub-watersheds in plain areas because of little topographic effects, i.e., one sub-watershed is taken as one contour band. According to the contour band, the whole Haihe basin is further discretized into 11,752 hydrologic response units (HRU) (Figure 5(b)). The details of the basin subdivision and coding are described in Luo et al. [57].

Table 1 shows a list of the main collected basic data on which the WEP-L input data are based. The data include the following categories: (1) hydro-meteorology; (2) land cover information including land use, vegetation, soil and water conservation, crop patterns, etc.; (3) topography, soil and hydrogeology; and (4) river network; etc. For data preparation and processing, it is referred to Jia et al. [29].

#### Model parameterization and sensitivity analysis

There are four categories of parameters in the WEP-L model: (1) parameters of land surface and river channel system; (2) parameters of vegetation; (3) parameters of soil and aquifer; and (4) parameters of snow melting. All of parameters are initially estimated according to land cover information, observation data, and remote sensing data. An explanation of estimation about some main parameters of these four categories referred to Jia et al. [29]. The disturbance analysis, a simple common method of sensitivity analysis, is used to analyze the sensitivities of main parameters and input data of the WEP-L model on the annual averages of model outputs. This method is that, when the model computes, one of the system parameter has an exiguity of change, while the other parameters are kept unchanged. Then the ratio of output change rate to the parameter change rate can be got, called as the sensitivity. The sensitivity analysis results indicates that the high sensitive include maximum depression storage of land surface, maximum soil moisture content (soil porosity), conductivity of river bed materials as well as thickness of soil layers. The middle sensitive include the conductivity and storage coefficient of aquifers, root depth and saturated conductivity of soils, etc. The low sensitive include vegetation parameters, manning roughness of river and lateral section shapes of river, etc.

#### Model calibration and validation

**Figure 6.**
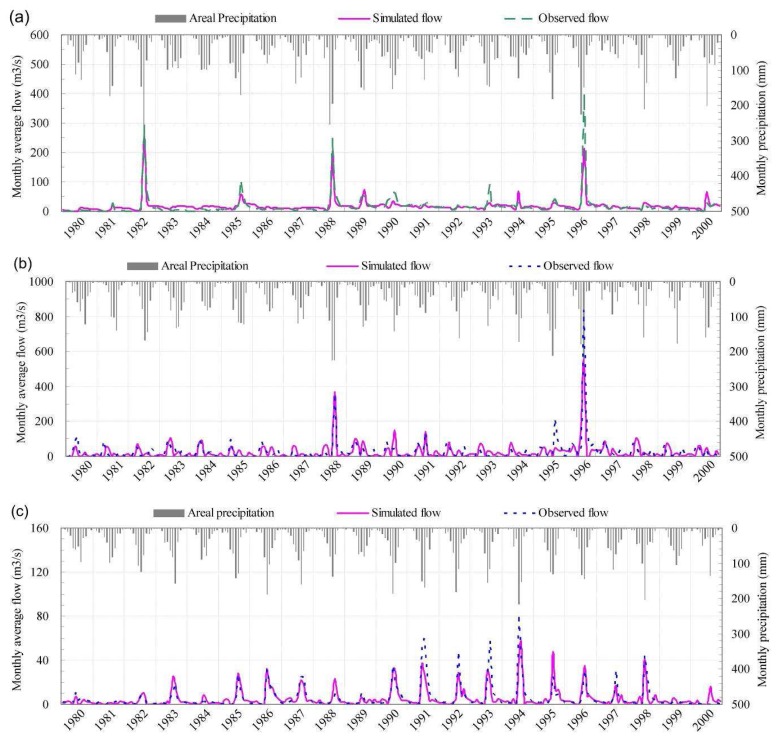

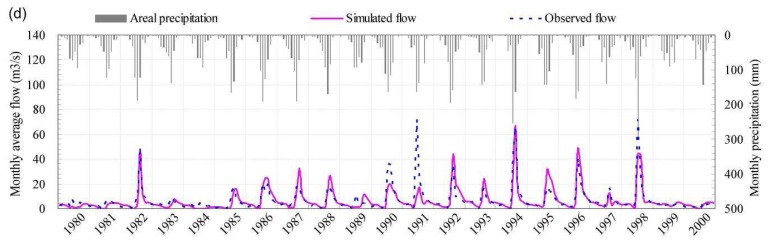
Validation of simulated monthly discharges at **(a)** Guantai station, **(b)** Huangbizhuang station, **(c)** Chengde station, and **(d)** Daiying station.

Sensitivity of parameters is analyzed, and then parameters with high sensitivity are calibrated on a basis of “try and error”. 11 years (1990-2000) is selected as calibration period. The calibration parameters include maximum depression storage depth of land surface, soil saturated hydraulic conductivity, hydraulic conductivity of unconfined aquifer, permeability of riverbed material, Manning roughness, snow melting coefficient, and critical air temperature for snow melting. After the model calibration, all parameters are kept unchanged, continuous simulations from 1980 to 2000 are performed to verify the model by using observed monthly discharges at 23 main gage stations in the basin. Simulation results of model indicate that average errors of annually runoff are less than 10%, Nash-Sutcliffe efficiency of monthly runoff at main gage stations is over 60%, and correlation coefficients between simulated and observed monthly runoff exceed 80%. A validation example at four stations is shown in Figure 6. Nash-Sutcliffe efficiency and multi-yearly errors of simulated monthly runoff at Guangtai, Huangbizhuang, Chengde and Daiying station are 0.4 and 6.5%, 0.68 and 5.3%, 0.72 and -0.6%, 0.66 and -3.0%, respectively.

### 4.2 SEBS application to the Haihe basin

In order to use SEBS algorithms, primary inputs required in this study are basically two folds: (1) Meteorological variables, such as, wind speed, air temperature, surface pressure, humidity and solar radiation; and (2) Remote sensing physical parameters derived from MODIS (Moderate-resolution Imaging Spectroradiometer) data onboard the Terra platform. The relevant parameters for this study are given in Table 2.

In this study the radiation, surface temperature, and surface vegetation properties are estimated from MODIS-Terra products [45, 51] for a detailed description of the input sources.. The MODIS sensors have a temporal resolution of a spatial resolution of 1×1 km. It is chosen because of its short revisit period and therefore higher chances to obtain cloud free images in the Haihe basin. The frequency of the MODIS-Terra data availability is once a day if it is cloud free, and the time of the satellite passing over the study area is around 11:00 a.m. local time. During the year 2005, totally 20 observations were available for the study area. Data preparation and processing are referred to in Shan [58].

The meteorological datasets were collected from the Chinese National Meteorological Center (NMC), which includes the standard meteorological observations over 50 meteorological stations in the Haihe River Basin on daily basis. Among these meteorological observations, relative humidity, wind speed, air temperature are measured on a hourly basis and are recorded every 6 hours at 2:00, 8:00, 14:00 and 20:00, while precipitation and sunshine hours are stored as daily values. A linear interpolation model is used to obtain the meteorological items at the satellite over passing time. The data is interpolated by ILWIS software to obtain the spatial meteorological items for the whole Haihe river basin.

**Figure 7.**
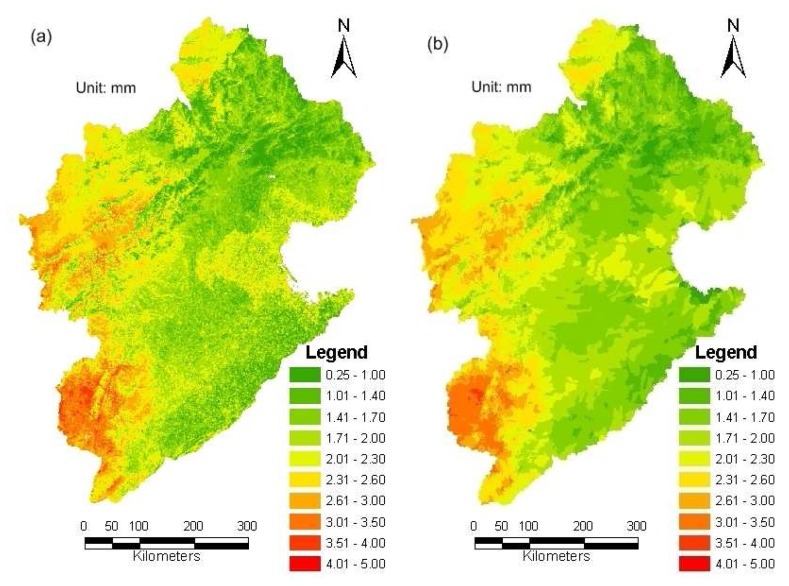
ETa distribution in September 17, 2005 as retrieved by the SEBS algorithm: **(a)** 1×1 km grid mode; and **(b)** Converted into 11752 HRUs.

SEBS first computes the net radiation flux using MODIS-L1B products and then estimates the ground heat flux following the empirical approach of Monteith [42] and Kustas and Daughtry [59]. Remote sensed estimates of surface thermal infrared emissivity and the reflectance of red and near infrared bands are used to compute spatial variations in reflected short-wave and emitted long-wave radiation away from the land surface. A combination of the short and long-wave radiation yields the possibility to compute the net radiation absorbed at the surface for every pixel. It is a key step to compute the sensible heat flux using turbulent heat equations by considering both the effective roughness height and aerodynamic resistance of the surface layer as well as the atmospheric stability of the boundary layer. For the latter, SEBS solves the similarity stability equations on momentum and potential temperature [45]. The latent heat flux, or equivalently the evapotranspiration, is then taken as the residual of the surface energy budget found by substracting the ground heat flux and sensible heat flux from the net radiation. Figure 7(a) shows an example of the distributions of daily actual evapotranspiration (ETa) calculated by SEBS for September 17, 2005.

### 4.3 Results and discussion of DA

The boundaries of the HRUs in WEP-L often differ from the boundaries of the satellite grids. In this study, pixel-to-pixel SEBS estimates are 1×1 km grid mode, whereas WEP-L predictions are given based on the 11752 HRUs. SEBS estimates are aggregated into 11752 HRUs (see Figure 7(b)) in order to make the data series matching for data assimilation. Although some spatial variability information could be missed in the process of data format conversion, SEBS ET distribution remains more spatially variable than model derived ET distribution. To update the WEP-L model simulated evapotranspiration, the model predicted actual evapotranspiration for the day with satellite coverage is used. Some obvious spatial differences between the simulated ET and remote sensing ET are found in Figs. 7(b) and 10 (a). This confirms the gap between the WEP-L model and SEBS retrievals. Although there is no definitive evidence to verify that the SEBS algorithm can provide better ET estimation than the WEP-L model, some studies on WEP-L show that its performance can be impacted due to the interferences of water diversions and reservoir regulations. This impact would be more obvious in the Haihe River basin, because over than 90% of water resources have been exploited in this region. ET simulation would be highly relative to water use information. So it would be difficult to attain the precise daily ET value with good spatial characteristics due to the difficulty to acquire the detailed water use process data. This would suggest that the assimilation of SEBS ET has the potential to improve the ET estimation.

**Figure 8.**
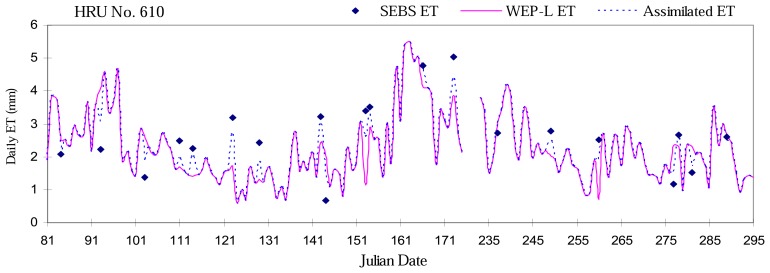
Time series plot of simulated ET with and without DA in a point HRU No. 610.

Different assimilation techniques existing correct the inputs, the internal states or the outputs of the models. The assimilation method used in our approach is a sequential method: the output states (ET) of the model are corrected using a weight-adaptive optimal interpolation algorithm based on the extended Kalman filter mentioned above. This methodology consists of locally correcting the value of the actual evapotranspiration of the WEP-L model when an observation is available. Figure 8 shows a time series plot of simulated ET with and without DA in a point HRU No. 610 (Sub-basin No.327, Contour band No.05). At the time step *k*, an observation is available. The difference between the observed (SEBS) and a priori simulated (WEP-L) values is partially corrected and an a posteriori value of the state, closer to the observed value is obtained. Then the WEP-L model continues and evolves freely until new observations are available. In order to avoid the water balance errors when correcting the states produced by the model, the assimilated ET would be constrained equal to the available water in the model (force mode) if the attained ET is still higher than the available water in the model after data assimilation.

**Figure 9.**
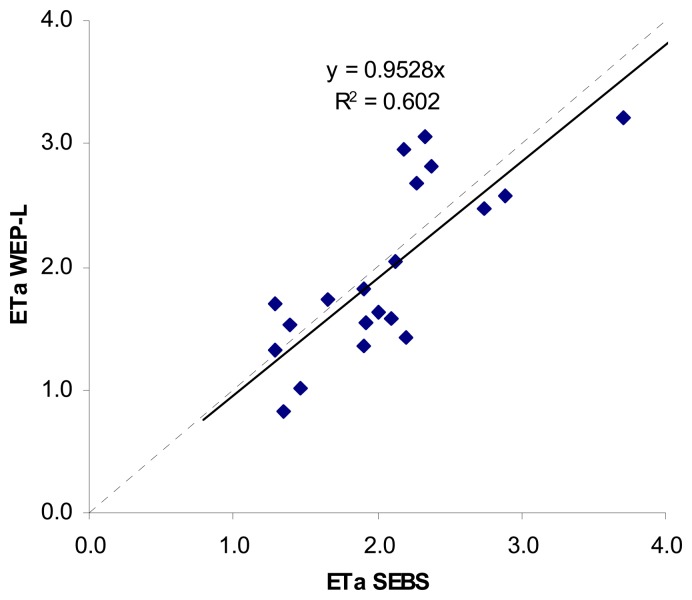
Scatter plot of the area averaged daily actual evapotranspiration in the case of available spectral data calculated by WEP-L and SEBS [mm]. The dashed line represents the 1 to 1 line, and the solid line the relationship between the data points from the WEP-L and SEBS.

**Figure 10.**
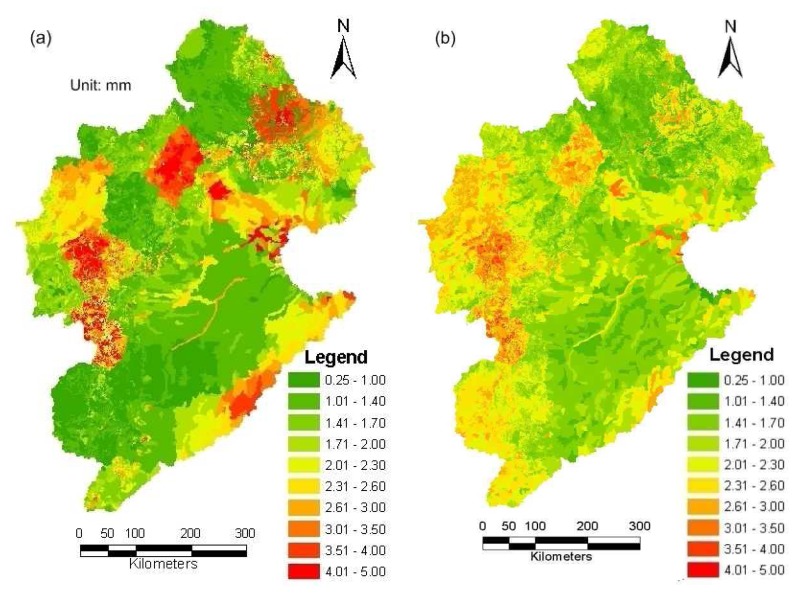
Comparison of ETa distribution in September 17, 2005 as calculated by the WEP-L model without and with data assimilation: **(a)** WEP-L ET; and **(b)** Assimilated ET.

A comparison between the area averaged daily actual evapotranspiration in the case of available MODIS spectral data by the WEP-L model and the SEBS algorithm is given in Figure 9. As can be seen from the figure, there is a good correlation (R2=0.60) between the daily ET estimated by SEBS and WEP-L. Although the basin averaged daily evapotranspiration show a good piecewise linear relationship between SEBS retrieval and WEP-L estimates in the basin level, their spatial distribution within the Haihe basin is different. Analyzing the SEBS and WEP-L results for 2005 (e.g., Figure 7(a) and Figure 10(a)), it is found that the remote sensing results contain more information about the spatial variability of the evapotranspiration estimates. Therefore, it is expected that above data assimilation system could lead to an improvement of the spatial water balance analysis using the WEP-L model.

Table 3 summarizes the results of the SEBS, WEP-L and assimilation respectively when averaged at the basin scale for the available day. Their spatial man, standard deviation and RMSE are given in this table. This RMSE between the observations and simulations is calculated as:

(12) $$\text{RMSE}=\sqrt{\frac{1}{n_{t}}\sum_{i}^{n_{t}}{\left(\theta_{o,i}-\theta_{s,i}\right)}^{2}}$$

*n_t_* is the number of data points, *θ_o_* the observed (SEBS) actual evapotranspiration, and *θ_s_* the simulated actual evapotranspiration. The RMSE can offer a measure of the “potential” from data assimilation. If the RMSE simulation with DA is lower than that of simulation without DA, this would means that the assimilation impact is positive. By the comparison of the RMSE of WEP-L derived actual evapotranspiration with and without DA, the conclusion is that data assimilation in this study leads to a positive impact on the WEP-L model. However, one must keep in mind that this is not sufficient for a definitive conclusion. This is because the SEBS derived actual evapotranspiration also contains errors.

**Figure 11.**
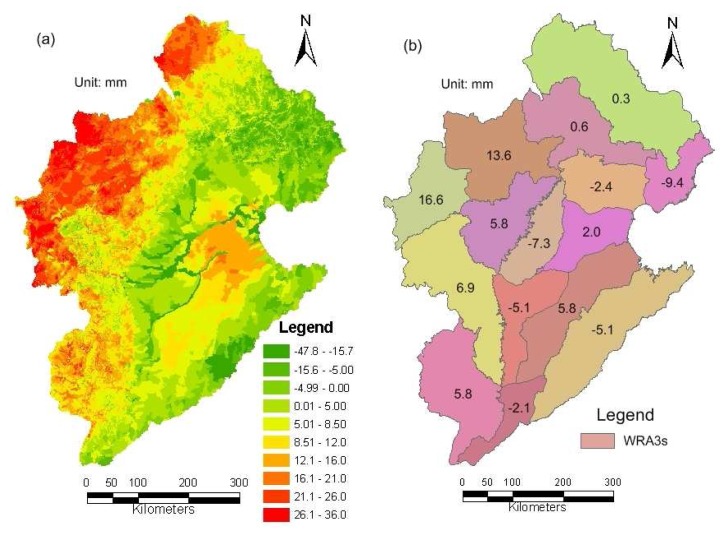
Spatially distributed difference in yearly evapotranspiration [mm] as calculated by the WEP-L model without and with data assimilation at the HRU level and WRA3 level, respectively: **(a)** HRU-level; and **(b)** WRA3 level. Negative values mean that the actual evapotranspiration calculated by the model with data assimilation is lower than that without data assimilation.

Figure 10(a) and (b) show an example about comparison of ETa distribution in September 17, 2005 as calculated by the WEP-L model without and with data assimilation. As can be seen in these figures, the assimilation procedure results contain more spatial variability than the WEP-L derived results without data assimilation. Figure 11(a) and (b) show the spatially distributed difference in annual evapotranspiration as calculated by the WEP-L model without and with data assimilation at the HRU and WRA3 level, respectively. Negative values in Fig11a and b mean that the actual evapotranspiration calculated by the model with data assimilation is lower than the actual evapotranspiration calculated without data assimilation. Figure 11(a) implies that SEBS estimates with limited daily remote sensing images yet clear spatial patterns appear in the assimilated ET distribution at the HRU level. The spatial differences of 11752 HRUs range from -48 to 32mm, and the largest relative difference can reach 16%. Figure 11(b) indicates that at the WRA3 level, the DA technique does not bring an obvious change to the yearly ET. Besides the WRA3-4 and WRA3-5, the spatial differences of most WRA3s range from -10 to 10mm, and the largest relative difference is less than ±1.5%. The spatial differences at the WRA3-4 and WRA3-5 are 16.6 and 13.6mm, and the relative differences are 4.2% and 3.7%, respectively.

Above analysis show that the assimilated ET does not differ much from the WEP-L ET at the WRA3 level, whereas the assimilated system makes more obvious spatial difference at the HRU-level. It is suggested that data assimilation system could at least remain its original accuracies of the WEP-L model at the higher scale, whereas more detailed description for spatial water balance can be given at the lower scale. Can we interpret these results? One possible reason is that the lack of detail in the database used to parameterize the hydrological states/fluxes is very likely to contribute to the incompatibility of the spatially distributed hydrological parameterization of the WEP-L model. Because although the whole basin is divided into 11752 hydrological response units in the model formulation, some parameters are given at a higher scale due to limited spatial information. This means that remote sensing observations have a potentially important role in providing spatial information to the assimilation system for the spatially optical hydrological parameterization of the model.

## 5. Conclusion and remarks

The paper has demonstrated the potential of remote sensing observations for updating the spatial water balance of distributed hydrological model. We have used an extended Kalman filter (EKF) as our data assimilation algorithm to handle this type of spatial information in the WEP-L model. By means of this data assimilation technique, estimates of daily evapotranspiration derived from MODIS-L1B products using the SEBS algorithm are combined with a distributed hydrological model (WEP-L) to understand the spatial distribution of water balance in a large basin with an area of 317,800 km^2^ in China. Assimilation results indicate that remote sensing observations have a potentially important role in providing spatial information to the assimilation system for the spatially optical hydrological parameterization of the model. This is especially important for large basins, such as the Haihe River basin in this study.

Both WEP-L and SEBS used in this study have robust physical mechanisms and their respective features. The WEP-L model is based on both water and energy balance and can provide temporally continuous hydrological simulation, whereas the SEBS algorithm is based on surface energy balance and can give more spatially variable information on surface energy fluxes. Assimilation of a combination of distributed hydrological model with available remote sensing data may provide the best spatial and temporal characteristics for hydrological states/fluxes. Therefore, combining and integrating the capabilities of and information from model simulation and remote sensing techniques would be both appealing and necessary for improving our knowledge of fundamental hydrological processes and for addressing important water resource management problems.

In the data assimilation experiment reported in this study, the benefits on the prediction precision of the model by assimilating MODIS-based ET need be further investigated, although RMSE results show data assimilation have a positive impact on the model. Unlike “synthetic” data assimilation experiments, there are no assumed “true” hydrologic states or fluxes for the DA system in this study. So it is difficult to evaluate to what extent the WEP-L prediction accuracy for the water balance can be improved by the integration of MODIS-based ET. So a key question, should be further investigated, is whether an energy balance model (such as SEBS) can provide better ET estimates than a distributed hydrological model (e.g. WEP-L).

A second key issue is whether more reliable spatial information can be achieved from remote sensing observations. For example, in this study only 20 cloud free MODIS images in the year 2005 can be attained for evapotranspiration retrieval. It remains challenging to retrieve energy fluxes under cloud cover. Another challenge is that the performance of the assimilation system for the combination of parameters optimization and state estimation should be further tested for the purpose of operational application.

## Figures and Tables

**Table 1.** List of collected basic data on which the model input based

Category	Item	Content
Meteorological and hydrology	Daily rain/snow	Data of 1502 rain stations, and 47 meteorological stations from 1956 to 2005
	Hourly rain/snow	Data of 47 rain stations from 1956 to 2005
	Wind speed	Daily data of 47 meteorological stations from 1956 to 2005
	Air temperature	Daily data of 47 meteorological stations from 1956 to 2005
	Sunshine hours	Daily data of 47 meteorological stations from 1956 to 2005
	Humidity	Daily data of 47 meteorological stations from 1956 to 2005
	Monthly runoff	Data of 23 hydrologic stations from 1956 to 2005

Remote sensing and land use	Landsat TM and deduces land use	1:100,000 map in 1986, 1996 and 2000
	NOAA-AVHRR	Monthly data between 1982 and 2000
	GMS	Monthly data between 1998 and 2002
	MODIS	Monthly data between 2002 and 2005

Vegetation	Vegetation fractional coverage	Deduced from NOAA-AVHRR from 1982 to2000 and MODIS from 2001 to 2005
	Leaf area index	Deduced from NOAA-AVHRR from 1982 to2000 and MODIS from 2001 to 2005
	Crop patterns	Data of 3rd level WRA districts in 1980, 1990 and 2000

Topography, soil and geohydrology	Topography	USGS GTOPO30 (1km by 1km DEM)
	Soil	1:1000,000 and 1:100,000 soil classification maps in China
	Geohydrology	Parameters of geohydrology, distribution of lithology and thickness of aquifers

River network	River network	River network map

Water use	Reservoir operation	Reservoir operation information of 44 reservoirs
	Water use in irrigation areas	Water use data in 75 irrigation area larger than 100 thousand mu
	Water use in administrative areas	Monthly water use data at the county level from 1956 to 2005
	Water diversion	Water diversion processes in representative districts

**Table 2.** Input parameters for SEBS.

Parameters	Estimation from

Surface temperature (°C)	MODIS derivative
Surface albedo (-)	MODIS derivative
NDVI (-)	MODIS derivative
DEM	MODIS derivative
PBL depth (m)	1000
Air temperature (°C)	50 meteorological stations
Relative humidity (kg/kg)	50 meteorological stations
Wind speed (m s^-1^)	50 meteorological stations
Surface pressure (Pa)	50 meteorological stations

**Table 3.** Comparison of spatial mean, standard deviation and RMSE of SEBS, WEP-L and assimilated actual evapotranspiration for the available day.

Julian date	Std. date	SEBS		WEP-L without DA			WEP-L with DA		
		
		Mean	St.dev	Mean	St.dev	RMSE	Mean	St.dev	RMSE
84	3/25	1.350	0.599	0.828	0.870	1.229	1.146	0.496	0.489
93	4/3	1.464	0.524	1.004	0.778	1.023	1.287	0.479	0.411
103	4/13	0.886	0.466	1.700	0.880	1.190	1.206	0.460	0.483
111	4/21	1.390	0.800	1.523	0.829	1.238	1.442	0.549	0.500
114	4/24	1.291	0.745	1.330	0.645	1.018	1.305	0.509	0.410
123	5/3	2.102	1.175	1.574	0.694	1.827	1.892	0.684	0.722
129	5/9	1.661	0.802	1.733	0.908	1.421	1.686	0.478	0.566
143	5/23	2.337	0.787	3.058	1.052	1.339	2.620	0.593	0.560
144	5/24	2.370	1.001	2.806	1.071	1.575	2.536	0.660	0.638
153	6/2	2.270	1.087	2.672	1.210	1.981	2.428	0.676	0.801
154	6/3	2.819	0.820	2.550	1.046	1.605	2.713	0.540	0.659
166	6/15	2.738	1.262	2.474	1.173	1.884	2.628	0.834	0.758
173	6/22	3.704	1.113	3.209	1.085	1.884	3.507	0.725	0.776
237	8/25	2.184	0.810	2.952	0.885	1.437	2.485	0.496	0.590
249	9/6	2.125	0.882	2.050	1.055	1.622	2.097	0.566	0.654
260	9/17	1.910	0.572	1.819	0.996	1.243	1.872	0.494	0.504
277	10/4	1.925	0.619	1.538	0.690	1.228	1.775	0.378	0.493
278	10/5	2.009	0.678	1.628	0.739	1.163	1.858	0.507	0.472
281	10/8	1.910	0.580	1.361	0.671	1.246	1.695	0.340	0.498
289	10/16	2.193	0.382	1.422	0.890	1.330	1.890	0.413	0.532
Mean		2.032	0.785	1.962	0.908	1.424	2.003	0.544	0.576
